# Characterization data of titanate compounds synthesized using hydrothermal method at various temperature

**DOI:** 10.1016/j.dib.2019.104992

**Published:** 2019-12-12

**Authors:** Mohd Hasmizam Razali, Nur Arifah Ismail, Uwaisulqarni M. Osman, Mohd Zul Helmi Rozaini, Mahani Yusoff

**Affiliations:** a School of Fundamental Science, Universiti Malaysia Terengganu, 21030, Kuala Nerus, Terengganu, Malaysia; b Institute of Biotechnology Marine, Universiti Malaysia Terengganu, 21030, Kuala Nerus, Terengganu, Malaysia; c Faculty of Bioengineering and Technology, Universiti Malaysia Kelantan Kampus Jeli, Karung Berkunci No. 100, 17600, Jeli, Kelantan, Malaysia

**Keywords:** Characterization, Titanate, Hydrothermal, Spectroscopy

## Abstract

Titanate compounds was synthesized using hydrothermal method at various temperature (100, 150, 200, and 250 °C) for 24 hours. As-synthesized titanate was characterized using FTIR, XRD and nitrogen gas adsorption. FTIR spectra was scanned from 4000 to 400 cm^−1^ using Perkin Elmer Spectrum 100 FTIR spectrophotometer. XRD diffractogram was performed by using Rigaku Miniflex (II) X-ray diffractometer operating at a scanning rate of 2.00° min^−1^. The diffraction spectra were recorded at the diffraction angle, 2θ from 10° to 80° at room temperature. Nitrogen gas adsorption analysis was studied by using Micromeritics ASAP2020 (Alaska) to determine the surface area and pores size distribution. The nitrogen adsorption and desorption was measured at 77 K (temperature of liquid nitrogen) and the samples were degassed in a vacuum at 110 °C under nitrogen flow for overnight prior to analysis.

**Table:** Specifications table

Subject	Chemistry, materials science
Specific subject area	Synthesis and characterization of materials
Type of data	Table Figure
How data were acquired	Data were acquired by FTIR, XRD and nitrogen gas adsorption.
Data format	Raw Analyzed
Parameters for data collection	FTIR and XRD was collected at room temperature. The nitrogen adsorption and desorption was measured at 77 K (temperature of liquid nitrogen) and the samples were degassed in a vacuum at 110 °C under nitrogen flow for overnight prior to analysis.
Description of data collection	FTIR spectra analysis was scanned from 4000 to 400 cm^−1^ using Perkin Elmer Spectrum 100 FTIR spectrophotometer XRD diffractogram was performed by using Rigaku Miniflex (II) X-ray diffractometer operating at a scanning rate of 2.00° min-1. The diffraction spectra were recorded at the diffraction angle, 2θ from 10° to 80° at room temperature. Nitrogen gas adsorption analysis was studied by using Micromeritics ASAP2020 (Alaska) to determine the surface area and pores size distribution.
Data source location	Universiti Malaysia Terengganu Kuala Nerus Malaysia
Data accessibility	With the article

**Table:** 

**Value of the Data** • Data obtained was important to study the physicochemical properties of materials • Data may be useful for future research • These data can support the performance of titanate compound

## 1 Data

**Fig. 1:**
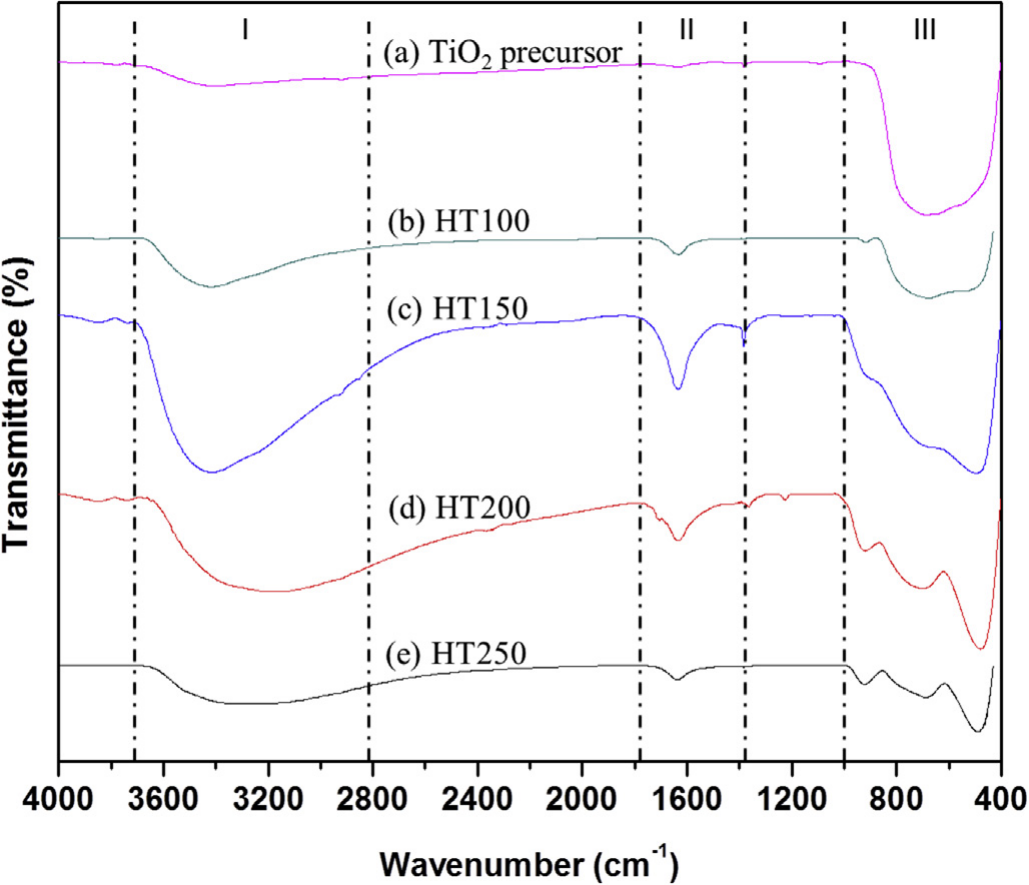
FTIR spectra of (a) TiO_2_ precursor, (b) HT100, (c) HT150, (d) HT200, and (e) HT250.

**Fig. 2:**
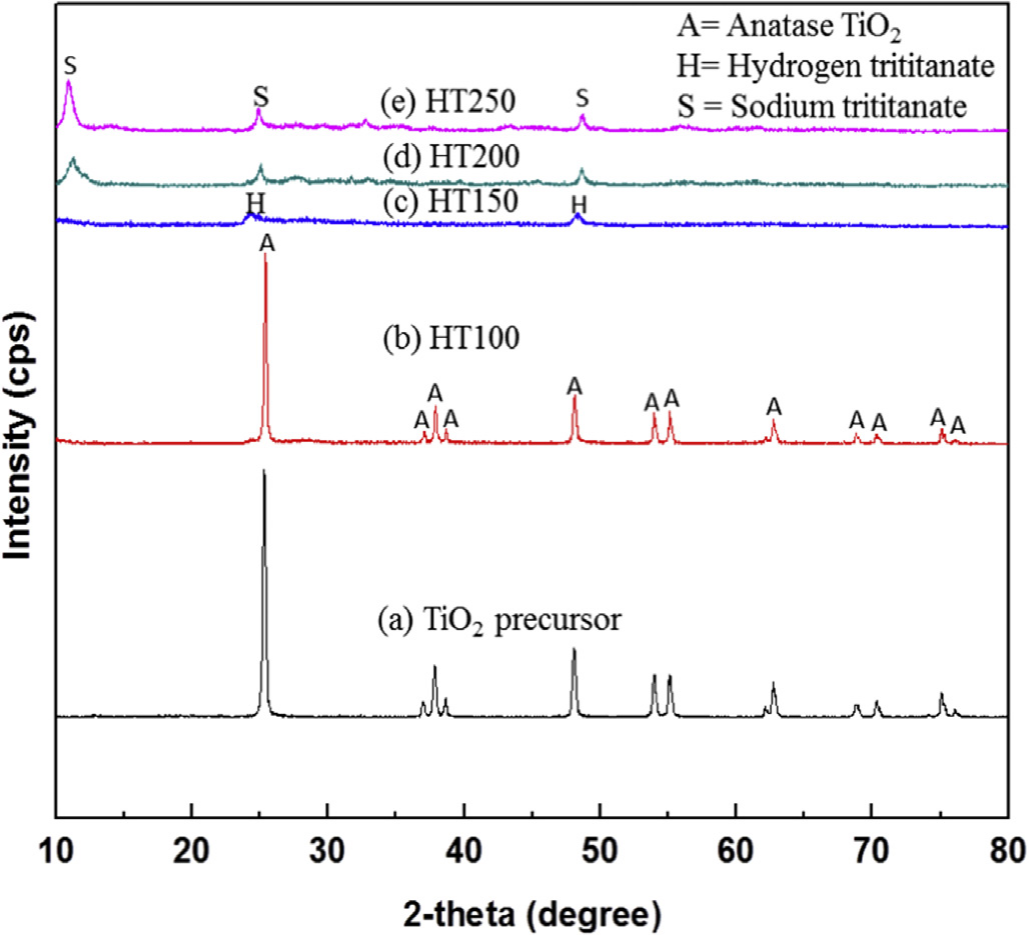
XRD diffractogram of (a) TiO_2_ precursor, (b) HT100, (c) HT150, (d) HT200, and (e) HT250.

**Fig. 3:**
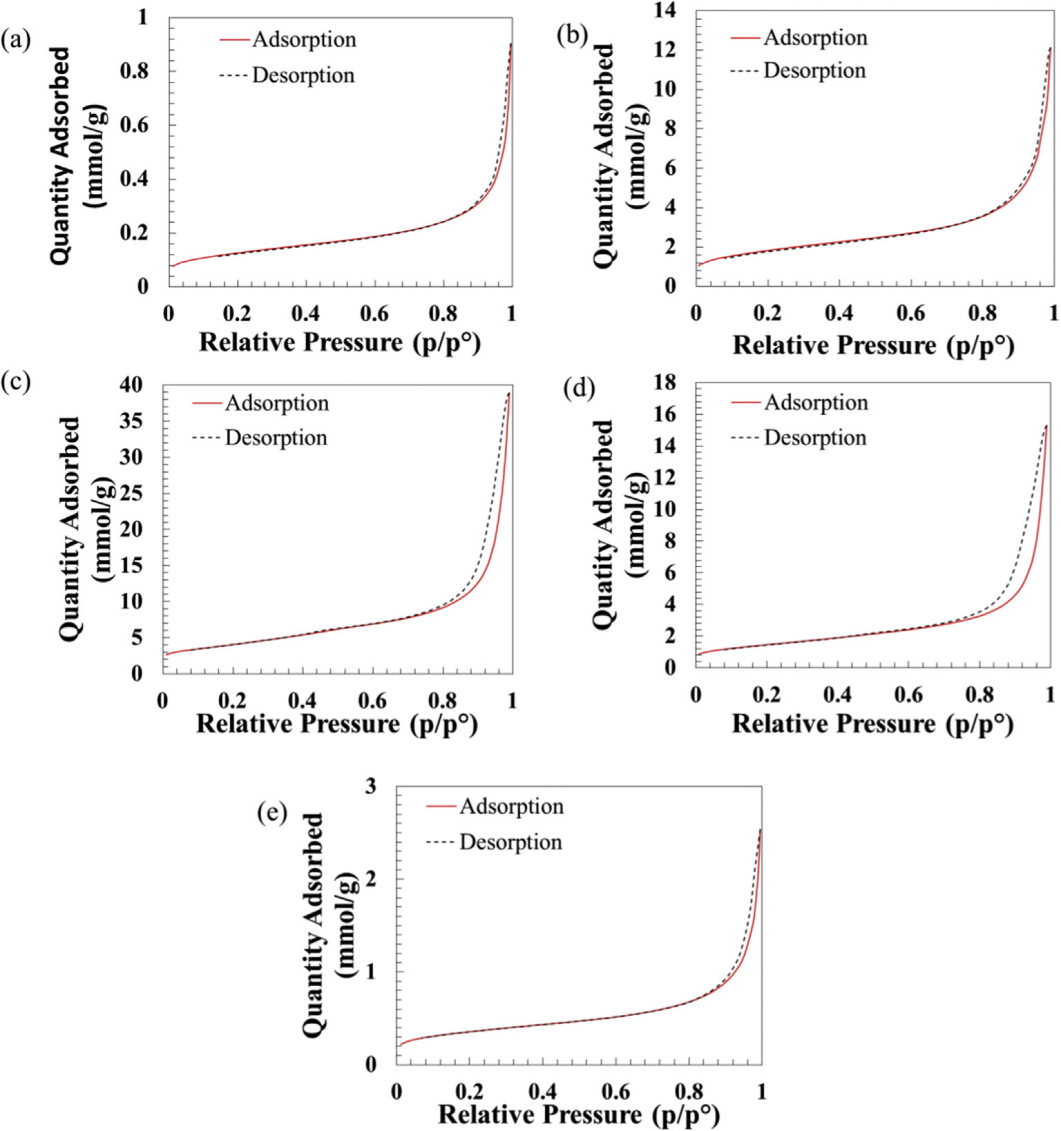
Nitrogen adsorption-desorption isotherms (a) TiO_2_ precursor, (b) HT100, (c) HT150, (d) HT200, and (e) HT250.

The dataset of this article provides information on characterization of titanate compounds produced using hydrothermal method at different temperature with commercial TiO_2_ powder was used as precursor. Fig. 1 shows the FTIR spectra of TiO_2_ precursor and as-synthesized titanate at 100, 150, 200, 250 °C hydrothermal temperature and their FTIR band assignment is presented in Table 1. The XRD patterns of the TiO_2_ precursor and as-synthesized titanate is shown in Fig. 2. While, Fig. 3 illustrates their N_2_ adsorption-desorption isotherm plot. The information about types of isotherms, hysteresis, pores and shape of pores as well as surface area, pore size and pore volume of the samples are tabulated in Table 1, Table 2, respectively.

**Table 1:** Assignment of FTIR bands for TiO_2_ precursor, HT100, HT150, HT200 and HT250.

Region	Wavelength (cm^−1^)	Assignment
I	3700–2800	OH stretching mode from water
		molecule
II	1800–1400	OH deformation mode from water
		molecule
III	<1000	Metal-oxygen stretching mode

**Table 2:** Types of isotherms, hysteresis, pores and shape of pores of TiO_2_ precursor, HT100, HT150, HT200, and HT250.

Samples	Type of isotherms	Type of hysteresis	Type of pores	Shape of pores
TiO_2_ precursor	IV	H3	Mesopore	Slit shaped pores
HT100	IV	H3	Mesopore	Slit shaped pores
HT150	IV	H3	Mesopore	Slit shaped pores
HT200	IV	H3	Mesopore	Slit shaped pores
HT250	IV	H3	Mesopore	Slit shaped pores

## 2 Experimental design, materials, and methods

2.0 g of TiO_2_ powder precursor (commercial TiO_2_ Merck) was dispersed in 10 M NaOH (100 ml) with constant stirring for 30 minutes (500 rpm). Then, the mixture was sonicated in sonicator bath for 30 minutes, after that continue with constant stirring for 30 minutes (500 rpm). Subsequently, the mixture was transferred into teflon vessel and subjected to hydrothermal treatment at various temperature (100, 150, 200, 250 °C) for 24 hours in autoclave. When the reaction was completed, the white solid precipitate was collected and dispersed into 0.1 M HCL (200 ml) with continuous stirring for 30 minutes (500 rpm) for washing. Then, the washing was followed by using a distilled water until the pH of washing solution was 7 and subsequently dried at 80 °C for 24 hours in an oven. As-synthesized sample at 100, 150, 200, and 250 °C hydrothermal temperature denoted as HT100, HT150, HT200, and HT250, respectively. The obtained samples were characterized using FTIR, XRD, and nitrogen gas adsorption.

Fig. 1 shows the FTIR spectra of TiO_2_ precursor and as-synthesized sample. A broad band has been observed in the range of 3700-2800 cm^−1^ and 1800-1400 cm^−1^. The metal-oxygen stretching mode has been detected below 1000 cm^−1^ attributed to the Ti–O bond. XRD analysis was carried out to study the phase structure of hydrothermally synthesized samples at different hydrothermal reaction temperature. For comparison the XRD pattern of TiO_2_ precursors was also included.

As can be seen in Fig. 2(a) and (b), the TiO_2_ precursor and as-synthesized sample at 100 °C (HT100) assigned to anatase TiO_2_ (JCPDS-ICDD No. 21–1272) [1]. Meanwhile for HT150, HT200 and HT250, their XRD is identical as hydrogen trititanate (JCPDS-ICDD No. 41–192) (Fig. 2 (c)) and sodium trititanate (JCPDS-ICDD No. 31–1392) (Fig. 2(d) and (e)), respectively [2]. These synthesized samples were assigned to trititanate compounds suggesting that the hydrothermal reaction of TiO_2_ precursor and NaOH occurs to produce titanate compounds.

Fig. 3 shows the N_2_ adsorption-desorption isotherm plot of the TiO_2_ precursor and as-synthesized samples at different hydrothermal temperature treatment. Table 2 shows the isotherm of studied samples exhibits a typical IV-like isotherm with H3 hysteresis [3]. Type IV isotherms is associated with capillary condensation in mesopore structures. While, the type H3 hysteresis represents slit shaped pores [4]. Table 3 possessed the BET surface area, pore size and pore volume of commercial TiO_2_ precursor powder and synthesized sample as different hydrothermal treatment.

**Table 3:** Surface area, pore size and pore volume of TiO_2_ precursor, HT100, HT150, HT200, and HT250.

Samples	Surface area (m^2^/g)	Pore size (nm)	Pore volume (cm^3^/g)
TiO_2_ precursor	10.07	12.36	0.03
HT100	146.74	11.29	0.43
HT150	320.51	14.93	1.36
HT200	117.51	16.32	0.53
HT250	28.15	14.90	0.09

As shown in Table 3, the surface area of commercial TiO_2_ precursor powder was only 10.07 m^2^/g. Nevertheless the surface area of the samples was increased after hydrothermal treatment. At 100 °C hydrothermal treatment (HT100), the surface area was found to be 146.74 m^2^/g. Meanwhile, HT150 which is the sample prepared at 150 °C hydrothermal treatment possessed the largest surface area (320.51 m^2^/g). The surface area of as-synthesized sample at 200 °C (HT200) is 117.51 m^2^/g and the surface area of HT250 sample was found to be only 28.15 m^2^/g. The pore sizes of the synthesized samples are between 11.29 to 16.32 nm, which is in the mesopore range and large pore volume from 0.03 to 1.36 were good for adsorbent.
